# Advantages of the single delay model for the assessment of insulin sensitivity from the intravenous glucose tolerance test

**DOI:** 10.1186/1742-4682-7-9

**Published:** 2010-03-18

**Authors:** Simona Panunzi, Andrea De Gaetano, Geltrude Mingrone

**Affiliations:** 1 CNR-Institute of Systems Analysis and Computer Science (IASI), BioMathLab, Rome, Italy; 2 Department of Internal Medicine, Catholic University, School of Medicine, Rome, Italy

## Abstract

**Background:**

The Minimal Model, (MM), used to assess insulin sensitivity (IS) from Intra-Venous Glucose-Tolerance Test (IVGTT) data, suffers from frequent lack of identifiability (parameter estimates with Coefficients of Variation (CV) less than 52%). The recently proposed Single Delay Model (SDM) is evaluated as a practical alternative.

**Methods:**

The SDM was applied to 74 IVGTTs from lean (19), overweight (22), obese (22) and morbidly obese (11) subjects. Estimates from the SDM (K_xgI_) were compared with the corresponding MM (S_I_), 1/HOMA-IR index and Euglycemic-Hyperinsulinemic Clamp (M-EHC over 7 subjects) estimates.

**Results:**

K_xgI _was identifiable in 73 out of 74 subjects (CV = 69% in the 74^th ^subject) and ranged from 1.25 × 10^-5 ^to 4.36 × 10^-4^min^-1^pM^-1^; S_I _CV was >52% in 36 subjects (up to 2.36 × 10^9^%) and presented 18 extreme values (≤ 1.5 × 10^-12 ^or ≥ 3.99).

K_xgI _correlated well with 1/HOMA-IR (r = 0.56, P < 0.001), whereas the correlations K_xgI_-S_I _and 1/HOMA-IR-S_I _were high (r = 0.864 and 0.52 respectively) and significant (P < 0.001 in both cases) only in the non-extreme S_I _sub-sample (56 subjects). Correlations K_xgI _vs. M-EHC and S_I _vs. M-EHC were positive (r = 0.92, P = 0.004 and r = 0.83, P = 0.02 respectively). K_xgI _decreased for higher BMI's (P < 0.001), S_I _significantly so only over the non-extreme-S_I _sub-sample. The Acute Insulin Response Index was also computed and the expected inverse (hyperbolic) relationship with the K_xgI _observed.

**Conclusions:**

Precise estimation of insulin sensitivity over a wide range of BMI, stability of all other model parameters, closer adherence to accepted physiology make the SDM a useful alternative tool for the evaluation of insulin sensitivity from the IVGTT.

## Background

Insulin Resistance (IR), an impaired metabolic response to circulating insulin resulting in a decreased ability of the body to respond to the hormone by suppressing Hepatic Glucose Output and enhancing tissue glucose uptake, plays a central role in the development of Type 2 Diabetes Mellitus. In fact, IR develops long before diabetes, as has been described in the relatives of type 2 diabetic patients [1]. Further, the metabolic consequences of elevated body mass index (BMI), such as IR, are the critical factors that confer risk for type 2 diabetes [2] or cardiovascular disease associated with fatness [3].

IR is present in a variety of diseases other than Type 2 Diabetes Mellitus and obesity, including hypertension [4], coronary heart disease [5], chronic renal failure [6], liver cirrhosis [7]. Due to the large prevalence of IR in the general population [8] and to its correlation and possibly causative role in many diseases [9], it has become of considerable interest to have an accurate measurement of the degree of IR by tests that are easy to perform and operator-independent. While the Euglycemic Hyperinsulinemic Clamp (EHC) has been long considered as the "golden standard" in clinical research [10], it requires careful training of the operator, and may be potentially dangerous for the subjects investigated due to the high levels of insulinemia reached during the test. Moreover, due to its intrinsic complexity (the subjects must lie in bed, infusion pumps and continuous bedside measurements of glycemia are required), this procedure is not easily applied to studies involving large patient samples. The Insulin Resistance Atherosclerosis Study (IRAS), for instance, performed on 398 black, 457 Hispanic, and 542 non-Hispanic white subjects, evaluated insulin sensitivity (S_I_) by the frequently sampled intravenous glucose tolerance test (IVGTT), analyzed by means of the Minimal Model (MM) [11]. The MM, introduced in the late seventies, also suffers, however, from some relevant problems, one of which is the frequent occurrence of "zero-S_I_" values, i.e. of very low point estimates of the insulin sensitivity index, particularly in large clinical studies [12].

Recently, on a series of subjects with BMI < 30 and with fasting glycemia < 7 mM [13], it was shown that the S_I _parameter from the MM is statistically unidentifiable (being estimated as not significantly different from zero) in as much as 50% of the healthy population. The possibility to reliably estimate an index of IR is, of course, crucial for any model aiming at being useful to diabetologists. Part of the problem of the lack of identifiability of the S_I _from the MM may reside in the MM being actually overparametrized with respect to the information available from the 23-point IVGTT [13]. Another important element determining this lack of identifiability resides in the parameter estimation strategy suggested by the proposing Authors [14] and commonly followed in applications, i.e. to use interpolated observed insulinemias (obviously affected by experimental error) as the input function in the model for fitting glycemias. This 'decoupling' fitting strategy delivers parameter estimates which optimize the adherence of the model to observed glycemias by considering random fluctuations of insulinemia as the true input signal: these estimates are, quite understandably, prone to error. In the recently published paper introducing the Single Delay Model (SDM) to assess insulin sensitivity after an IVGTT [13], the effect of avoiding the above sources of error is discussed in detail.

The appropriate mathematical behaviour of the SDM itself has also been the object of a previous paper [15]. The SDM was designed to fit simultaneously both glucose and insulin time courses with a reduced number of parameters (six free parameters overall instead of at least eight for the MM if both glycemias and insulinemias are predicted), and was shown to provide robust and precise estimates of insulin sensitivity in a sample of non-obese subjects with normal fasting glycemia.

The goal of the present study is to apply the same SDM to a heterogeneous population, consisting of overweight, obese and morbidly obese subjects compared with lean individuals, in order to verify the performance of this model over the entire BMI range of interest for diabetologists.

## Methods

### Experimental protocol

Data related to 74 healthy volunteers and obese subjects (28 males, 46 females, BMI from 18.51 to 62.46 [Kg/m^2^], average anthropometric characteristics reported in Table 1) from archived, unpublished studies conducted at the Catholic University Department of Metabolic Diseases in Rome, were analyzed.

**Table 1:** Anthropometric characteristic of the studied subjects along with the descriptives of the 1/HOMA-IR and HOMA2 indices and of the two insulin-sensitivity indices K_xgI _and S_I_in the Full Sample and in the Sub-sample (not including extreme S_I_values)

		Anthropometric characteristic Full Sample					
		
		Age	Height (cm)	BW (Kg)	BMI	G_b_ (mM)	I_b_ (pM)
**BMI ≤ 24**	**Mean**	41.7	166.8	62.7	22.4	4.4	33.0
	
	**Std. Dev**.	18.5	9.8	9.5	1.7	0.6	13.2
	
	**Std. Err**.	4.2	2.2	2.2	0.4	0.1	3.0
	
	**N**	19	19	19	19	19	19

**24>BMI ≤ 30**	**Mean**	47.2	166.0	71.3	25.8	4.6	46.1
	
	**Std. Dev**.	14.8	7.9	8.8	1.3	0.5	26.5
	
	**Std. Err**.	3.2	1.7	1.9	0.3	0.1	5.6
	
	**N**	22	22	22	22	22	22

**30>BMI ≤ 40**	**Mean**	49.5	163.0	91.5	34.3	4.3	70.0
	
	**Std. Dev**.	17.5	8.3	12.4	2.7	0.5	46.4
	
	**Std. Err**.	3.7	1.8	2.6	0.6	0.1	9.9
	
	**N**	22	22	22	22	22	22

**BMI>40**	**Mean**	40.4	162.0	127.4	48.7	4.8	96.4
	
	**Std. Dev**.	9.7	8.4	16.2	6.7	0.4	59.7
	
	**Std. Err**.	2.9	2.5	4.9	2.0	0.1	18.0
	
	**N**	11	11	11	11	11	11

**Total**	**Mean**	45.5	164.7	83.4	30.9	4.5	57.3
	
	**Std. Dev**.	16.2	8.6	24.3	9.3	0.5	42.7
	
	**Std. Err**.	1.9	1.0	2.8	1.1	0.1	5.0
	
	**N**	74	74	74	74	74	74

		**Full Sample**					
		
		**1/HOMA-IR**		**HOMA2**		**K_xgI_**	**S_I_**

**BMI ≤ 24**	**Mean**	1.4		1.64		1.6E-04	47.2
	
	**Std. Dev**.	1.1		0.51		9.3E-05	205.8
	
	**Std. Err**.	0.3		0.13		2.1E-05	47.2
	
	**N**	19		16		19	19

**24>BMI ≤ 30**	**Mean**	1.0		1.37		1.3E-04	13.8
	
	**Std. Dev**.	0.6		0.59		7.6E-05	64.6
	
	**Std. Err**.	0.1		0.13		1.6E-05	13.8
	
	**N**	22		20		22	22

**30>BMI ≤ 40**	**Mean**	0.8		1.16		8.4E-05	101.3
	
	**Std. Dev**.	0.4		0.67		7.1E-05	246.9
	
	**Std. Err**.	0.1		0.14		1.5E-05	52.6
	
	**N**	22		22		22	22

**BMI>40**	**Mean**	0.4		0.73		2.8E-05	139.8
	
	**Std. Dev**.	0.2		0.30		9.5E-06	270.9
	
	**Std. Err**.	0.1		0.09		2.9E-06	81.7
	
	**N**	11		11		11	11

**Total**	**Mean**	1.0		1.26		1.1E-04	67.1
	
	**Std. Dev**.	0.8		0.62		8.5E-05	203.3
	
	**Std. Err**.	0.1		0.08		9.9E-06	23.6
	
	**N**	74		69		74	74

		**Sub-Sample**					
		
		**1/HOMA-IR**		**HOMA2**		**K_xgI_**	**S_I_**

**BMI ≤ 24**	**Mean**	1.5		1.68		1.6E-04	1.4E-04
	
	**Std. Dev**.	1.1		0.53		9.6E-05	8.9E-05
	
	**Std. Err**.	0.3		0.15		2.4E-05	2.2E-05
	
	**N**	16		13		16	16

**24>BMI ≤ 30**	**Mean**	1.0		1.40		1.3E-04	1.1E-04
	
	**Std. Dev**.	0.6		0.59		7.8E-05	6.3E-05
	
	**Std. Err**.	0.1		0.14		1.7E-05	1.4E-05
	
	**N**	21		19		21	21

**30>BMI ≤ 40**	**Mean**	0.6		0.98		5.3E-05	7.5E-05
	
	**Std. Dev**.	0.4		0.68		2.8E-05	7.8E-05
	
	**Std. Err**.	0.1		0.21		8.5E-06	2.4E-05
	
	**N**	11		11		11	11

**BMI>40**	**Mean**	0.4		0.70		2.8E-05	3.6E-05
	
	**Std. Dev**.	0.2		0.30		1.0E-05	1.4E-05
	
	**Std. Err**.	0.1		0.11		3.6E-06	4.8E-06
	
	**N**	8		8		8	8

**Total**	**Mean**	1.0		1.27		1.1E-04	1.0E-04
	
	**Std. Dev**.	0.8		0.65		8.5E-05	7.8E-05
	
	**Std. Err**.	0.1		0.09		1.1E-05	1.0E-05
	
	**N**	56		51		56	56

19 subjects were lean individuals (BMI ≤ 24 Kg/m^2^, average 22.40 ± 1.68 SD), 22 were overweight (24< BMI ≤ 30 Kg/m^2^, average 25.78 ± 1.34), 22 were obese (30 < BMI ≤ 40 Kg/m^2^, average 34.34 ± 2.74) and 11 were morbidly obese (BMI > 40 Kg/m^2^, average 48.68 ± 6.68). All subjects had negative family and personal histories for Diabetes Mellitus and other endocrine diseases, were on no medications, had no current illness and had maintained a constant body weight for the six months preceding each study.

For the three days preceding the study each subject followed a standard composition diet (55% carbohydrate, 30% fat, 15% protein) ad libitum with at least 250 g carbohydrates per day. Written informed consent was obtained in all cases; all original study protocols were conducted according to the Declaration of Helsinki and along the guidelines of the institutional review board of the Catholic University School of Medicine, Rome, Italy.

Each study was performed at 8:00 AM, after an overnight fast, with the subject supine in a quiet room with constant temperature of 22-24°C. Bilateral polyethylene I.V. cannulas were inserted into antecubital veins. The standard IVGTT was employed (without either Tolbutamide or insulin injections) [11]: at time 0 (0') a 33% glucose solution (0.33 g Glucose/kg Body Weight) was rapidly injected (less than 3 minutes) through one arm line. Blood samples (3 ml each, in lithium heparin) were obtained at -30', -15', 0', 2', 4', 6', 8', 10', 12', 15', 20', 25', 30', 35', 40', 50', 60', 80', 100', 120', 140', 160' and 180' through the contralateral arm vein. Each sample was immediately centrifuged and plasma was separated. Plasma glucose was measured by the glucose oxidase method (Beckman Glucose Analyzer II, Beckman Instruments, Fullerton, CA, USA). Plasma insulin was assayed by standard radio immunoassay technique. The plasma levels of glucose and insulin obtained at -30', -15' and 0' were averaged to yield the baseline values referred to 0'.

Seven out of the 74 subjects also underwent a Hyperinsulinemic-Euglycemic glucose Clamp study. They were admitted to the Department of Metabolic Diseases at 6.00 p.m. of the day before the study. At 7:00 a.m. on the following morning, indirect calorimetric monitoring was started; the infusion catheter was inserted into an antecubital vein; the sampling catheter was introduced in the contralateral dorsal hand vein and this hand was kept in a heated box (60°C) to obtain arterialized blood. The glycemia of diabetic patients was maintained below 100 mg/dl by small bolus doses of short-acting human insulin (Actrapid HM, Novo Nordisk, Denmark) until the beginning of the study. At 9.00 a.m., after 12 to 14 hour overnight fast, the euglycemic hyperinsulinemic glucose clamp was performed as described by De Fronzo et al [16]. A priming dose of short-acting human insulin was given during the initial 10 minutes in a logarithmically decreasing way, in order to acutely raise the serum insulin to the desired concentration. Insulin concentration was then maintained approximately constant with a continuous infusion of insulin at an infusion rate of 40 mIU/m^2^/minute for 110 minutes.

### The Single Delay Model (SDM)

**Figure 1:**
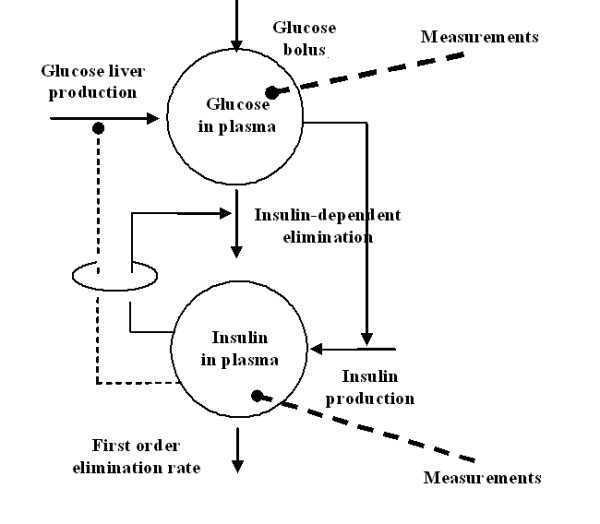
**Block diagram of the Single Delay Model**. The model consists of two compartments: the glucose plasma concentrations and the insulin plasma concentrations. Elimination of glucose from plasma occurs depending on plasma insulin concentrations.

The schematic diagram of the mathematical model is represented in Figure 1 and its equations are reported below:(1)

The meaning of the structural parameters is reported in Table 2. The initial condition G_b_+GΔ expresses the glucose concentration as variation with respect to the basal conditions, as a consequence of the I.V. glucose bolus. In equation (2), the second term represents second-phase insulin delivery from the β-cells. Its functional form is consistent with the hypothesis that insulin production is limited, reaching a maximal rate of release T_igmax_/V_i _by way of either a Michaelis-Menten dynamics or a sigmoidal shape, according to whether the γ value is 1 or greater than 1 respectively. Situations where γ is equal to zero correspond to a lack of response of the pancreas to variations of circulating glucose, while for γ values between zero and 1 the shape of the response resembles a Michaelis-Menten, with a sharper curvature towards the asymptote. The parameter γ expresses therefore the capability of the pancreas to accelerate its insulin secretion in response to progressively increasing blood glucose concentrations. The initial condition I_b_+IΔ_G_GΔ represents the immediate first-phase response of the pancreas to the sudden increment in glucose plasma concentration. The model is discussed in detail in [13].

**Table 2:** Definition of the symbols used in the discrete Single Delay Model

Symbol	Units	Definition
G(t)	[mM]	glucose plasma concentration at time t

G_b_	[mM]	basal (preinjection) plasma glucose concentration

I(t)	[pM]	insulin plasma concentration at time t

I_b_	[pM]	basal (preinjection) insulin plasma concentration

K_xgI_	[min^-1 ^pM^-1^]	net rate of (insulin-dependent) glucose uptake by tissues per pM of plasma insulin concentration

T_gh_	[mmol min^-1 ^kgBW^-1^]	net balance of the constant fraction of hepatic glucose output (HGO) and insulin-independent zero-order glucose tissue uptake

V_g_	[L kgBW^-1^]	apparent distribution volume for glucose

D_g_	[mmol kgBW^-1^]	administered intravenous dose of glucose at time 0

G_Δ_	[mM]	theoretical increase in plasma glucose concentration over basal glucose concentration at time zero, after the instantaneous administration and distribution of the I.V. glucose bolus

K_xi_	[min^-1^]	apparent first-order disappearance rate constant for insulin

T_igmax_	[pmol min^-1 ^kgBW^-1^]	maximal rate of second-phase insulin release; at a glycemia equal to G* there corresponds an insulin secretion equal to T_igmax_/2

V_i_	[L kgBW^-1^]	apparent distribution volume for insulin

τ_g_	[min]	apparent delay with which the pancreas changes secondary insulin release in response to varying plasma glucose concentrations

γ	[#]	progressivity with which the pancreas reacts to circulating glucose concentrations. If γ were zero, the pancreas would not react to circulating glucose; if γ were 1, the pancreas would respond according to a Michaelis-Menten dynamics, with G* mM as the glucose concentration of half-maximal insulin secretion; if γ were greater than 1, the pancreas would respond according to a sigmoidal function, more and more sharply increasing as γ grows larger and larger

I_ΔG_	[pM mM^-1^]	first-phase insulin concentration increase per mM increase in glucose concentration at time zero due to the injected bolus

G*	[mM]	glycemia at which the insulin secretion rate is half of its maximum

From the steady state condition at baseline it follows that:

The index of insulin sensitivity is easily derived from this model by applying the same definition as for the Minimal Model [11], i.e.(3)

and coincides therefore with one of the model structural parameters to be estimated. It is expressed in the same units of measurement as the MM-derived S_I _index (min^-1 ^pM^-1^) [13].

### Insulin Sensitivity determination with the SDM

For each subject the discrete Single Delay Model [13] was fitted to glucose and insulin plasma concentrations by Generalized Least Squares [17], in order to obtain individual regression parameters along with an estimate for the glucose and insulin coefficients of variation. All observations on glucose and insulin were considered in the estimation procedure except for the basal levels. Coefficients of variation (CV) for glucose and insulin were estimated in phase 2 of the GLS algorithm, whereas single-subject CVs for the model parameter estimates were derived from the corresponding estimated asymptotic variance-covariance matrix of the GLS estimators.

### Insulin Sensitivity determination with the MM

For the MM, fitting was performed by means of a Weighted Least Squares (WLS) estimation procedure, considering as weights the inverses of the squares of the expectations and as coefficient of variation for glucose 1.5% [14]. Observations on glucose before 8 minutes from the bolus injection, as well as observations on insulin before the first peak were disregarded, as suggested by the proposing Authors [11,18]. A BFGS quasi-Newton algorithm was used for all optimizations [19]. The insulin sensitivity index was computed as the ratio between the MM parameters *p*_3 _and *p*_2 _representing respectively the scale factor governing the amplitude of insulin action, and the elimination rate constant of the remote insulin compartment were insulin action takes place.

### Basal insulin sensitivity measurements and HOMA

Studies conducted in a population of overweight and obese postmenopausal women [20] and in polycystic ovary syndrome and menopausal patients [21] have demonstrated that surrogate measures of insulin resistance, as for example the HOMA index, the fasting insulin, the QUICKY index etc, are simple tools, appropriate in large sample studies, that can be used as substitutes for the EH clamp. In this study the HOMA, though simplistic and approximate tools for a real assessment of insulin sensitivity, was therefore used to perform comparisons and assess coherence among the model derived indices, as the EHC-derived M was not available for most of the evaluated subjects.

The HOMA insulin resistance index was computed as the product of the fasting values of glucose, expressed as mM, and insulin, expressed as μIU/mL, divided by the constant 22.5) [22-24]. Its reciprocal 1/HOMA-IR [25], was used as insulin sensitivity index. The HOMA2 insulin sensitivity index was obtained by the program HOMA Calculator v2.2.2 [26].

### Statistical analysis

Model fitting was performed using Matlab version 7 (The MathWorks, Inc) whereas statistical analyses were performed using R (version 2.6.1 Copyright 2007 The R Foundation for Statistical Computing). The entire sample composed of 74 subjects was divided into four groups: lean subjects (BMI less or equal to 24), overweight subjects (BMI between 24 and 30), obese (BMI greater than 30 and less or equal to 40) and morbidly obese subjects (BMI greater than 40). For each parameter of the SDM and MM the a-posteriori model identifiability was determined by computing the asymptotic coefficients of variation for the free model parameters: a CV smaller than 52% translates into a standard error of the parameter smaller than 1/1.96 of its corresponding point estimate and into an asymptotic normal confidence region of the parameter not including zero.

One-way ANOVAs were performed to determine if a significant difference arose among the four groups for the variables K_xgI_, S_I_, 1/HOMA-IR and HOMA2.

The different insulin sensitivity indices were correlated using Pearson's r coefficient.

A further comparison was made between the insulin sensitivity (M index) assessed with Euglycemic Hyperinsulinemic Clamp and either of the two model-derived insulin sensitivity indices (K_xgI _and S_I_) on the 7 subjects who underwent both IVGTT and EHC. Given the small number of subjects, both the parametric Pearson's r correlation coefficient and the nonparametric Spearman coefficient were computed.

## Results

### SDM and MM fitting

**Figure 2:**
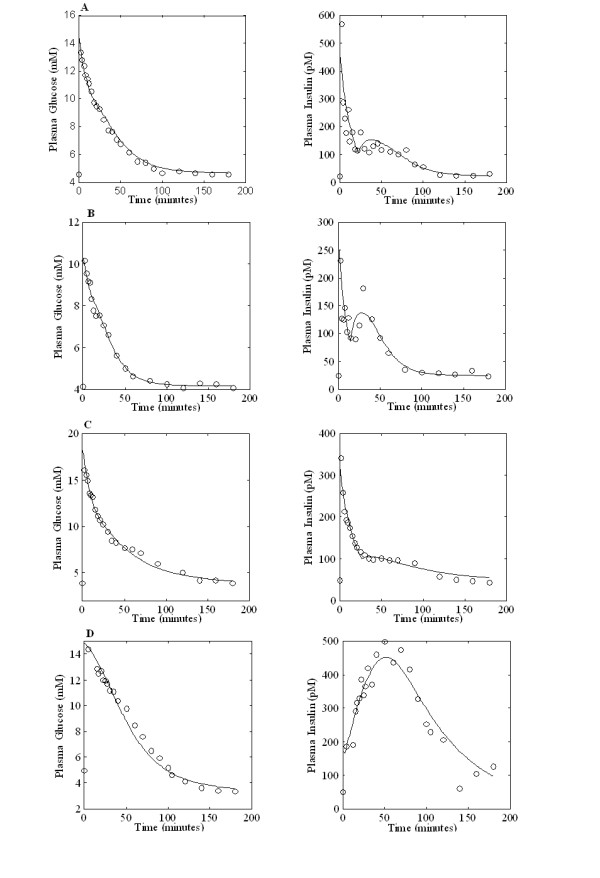
**Glucose and Insulin observed concentrations (circles) along with their Single Delay Model time predictions (continuous line) for four subjects belonging to different BMI classes**. Panel A: one subject with BMI ≤ 24, Panel B: one subject with 24 < BMI ≤ 30, Panel C: one subject with 30 < BMI ≤ 40, Panel D: one subject with BMI > 40

**Figure 3:**
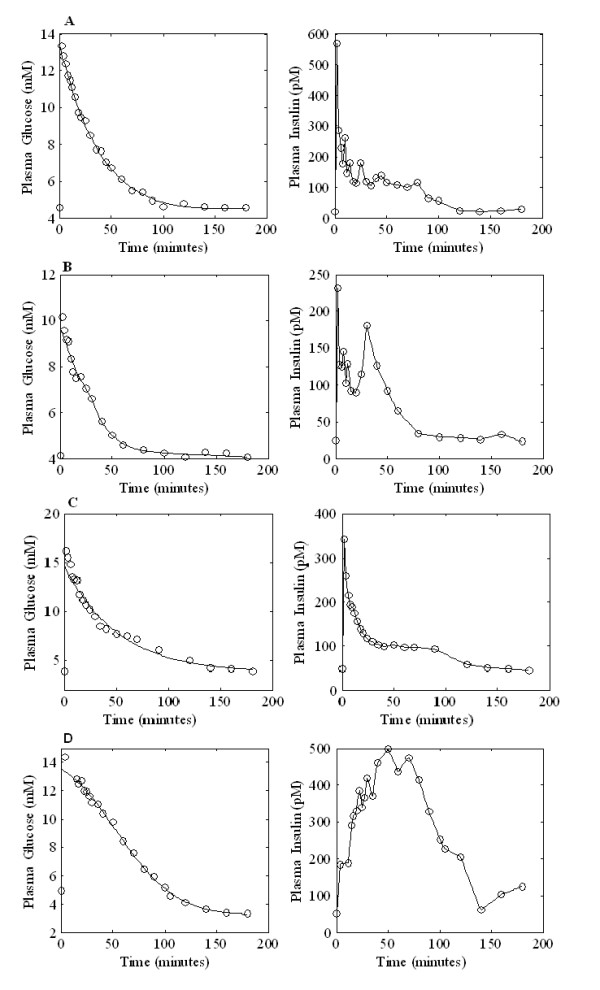
**Glucose and Insulin observed concentrations (circles) along with the Minimal Model glucose time predictions and interpolated insulin observations (continuous line) for four subjects belonging to different BMI classes**. Panel A: one subject with BMI ≤ 24, Panel B: one subject with 24 < BMI ≤ 30, Panel C: one subject with 30 < BMI ≤ 40, Panel D: one subject with BMI > 40.

The two models were both able to satisfactorily fit all the available data sets (but see discussion in [13]). Figure 2 shows the experimental data of glucose and insulin concentrations as well as the corresponding time course predictions from the SDM for four subjects, each from one of the four different BMI subgroups. Figure 3 shows the same four subjects fitted with the MM. In this case only glucose concentrations were fitted, whereas insulin observations were linearly interpolated as the MM Authors suggest.

The sensitivity index K_xgI _from the SDM was identifiable (CV < 52%) in 73 out of 74 subjects. For the remaining subject the CV was equal to 68.83% (K_xgI _= 2.87 × 10^-4^).

The sensitivity index S_I _from the MM was not identifiable (CV ≥ 52%) in 36 subjects out of 74, where coefficients of variation ranged from 52.76% to 2.36 × 10^+9 ^%. In 18 of these subjects the S_I _estimates were either suspiciously large (from 3.99 to 890 in 11 subjects) or very small (less than or equal to 1.5 × 10^-12^, the so called "zero-S_I_", in 7 subjects).

### Comparison between K_xgI_, S_I_, 1/HOMA-IR and HOMA2

The relationship between the four indices was examined by means of the Pearson correlation coefficient. Two situations were examined, either considering the entire 74-subject sample (the "whole sample"), or considering a sub-sample (the "reduced sub-sample") obtained by eliminating those 18 subjects whose S_I _values were extreme (11 very large, > 3; 7 very small, = 1.5 × 10^-12^). The computation of HOMA2 was not performed for 5 subjects whose basal insulin values were below 20 pmol. No of these subjects presented extreme S_I _values.

The correlations between K_xgI _and 1/HOMA-IR and between K_xgI _and HOMA2 were positive and highly significant both in the whole sample (r = 0.565, P < 0.001 and r = 0.581, P < 0.001 respectively) and in the reduced sub-sample (r = 0.572, P < 0.001 and r = 0.558, P < 0.001 and respectively).

The correlations between S_I _and 1/HOMA-IR and between S_I _and HOMA2 were positive and significant (r = 0.525, P < 0.001 and r = 0.454, P = 0.001 respectively) only when the reduced sub-sample was considered, whereas in the whole sample no correlation was apparent (r = -0.074, P = 0.529 and r = 0.015, P = 0.904 respectively).

**Figure 4:**
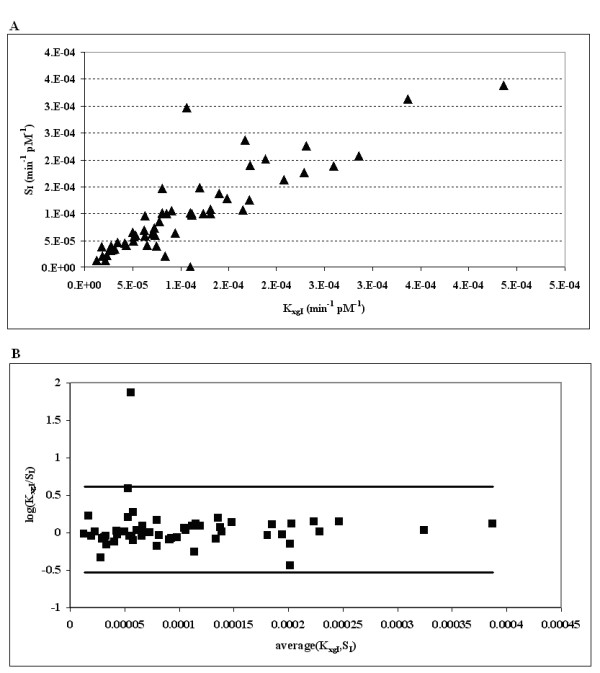
**Panel A: scatter plot of the two Insulin Sensitivity Indices from the Single Delay Model (K_xgI_) and from the Minimal Model (S_I_) on the reduced Sub-Sample obtained eliminating the 18 extreme-S_I _subjects**. Panel B: Bland-Altman Procedure; on the abscissas are reported the averages of each pair of Insulin Sensitivity Indices (one from the Single Delay Model K_xgI _and one from the Minimal Model S_I_) from the reduced Sub-Sample (obtained eliminating the 18 extreme-S_I _subjects); on the ordinates are reported the logarithms of the ratios between each subject's K_xgI _and S_I_.

In the reduced sub-sample, where the extreme-S_I _subjects are not considered, correlation between K_xgI _and S_I _was clearly positive and significant (r = 0.864, P < 0.001), see Panel A of Figure 4. In this reduced sub-sample, absolute values also agreed very well (mean K_xgI _= 1.07 × 10^-4 ^vs. mean S_I _= 1.01 × 10^-4^).

The results of a Bland-Altman procedure on K_xgI _and S_I _are reported in Panel B of Figure 4. Because of the non-uniformity of the variance (the differences between each pair of insulin sensitivity indices depend on the values of the computed indices), the logarithms of the ratios instead of absolute differences are reported on the ordinates. The 95% interval around the average mean is reported along with the individual points. From an inspection of the graph it can be easily seen that, in the sub-sample without extreme S_I _values, the two methods are equivalent. An equivalent Bland-Altman procedure could not be performed on the whole sample, given the extreme values attained by the MM-derived S_I_.

### Comparison between the four BMI-classes

**Figure 5:**
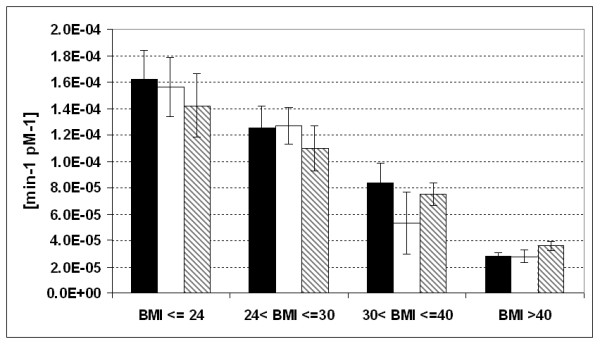
**Mean values and standard errors for the Insulin Sensitivity Indices from the Single Delay Model (K_xgI_) and from the Minimal Model (S_I_)**. For the K_xgI _the average values were computed both in the Full Sample and in the reduced Sub-Sample. The average values of the S_I _index over the Full Sample were out of scale for all four groups and could not be plotted. Black bar = K_xgI _in the Full Sample, white bar = S_I _in the reduced Sub-Sample, striped bar = K_xgI _in the reduced Sub-Sample. Post-Hoc analysis by LSD test: for the K_xgI _in the Full Sample the significant comparisons were 1 vs 3 (P = 0.001), 1 vs 4 (P < 0.001), 2 vs 4 (0.001) and 3 vs 4 (P = 0.047); for the K_xgI _in the reduced Sub-Sample the significant comparisons were: 1 vs 3 (P = 0.001), 1 vs 4 (p < 0.001), 2 vs 3 (P = 0.008), 2 vs 4 (P = 0.002); for the S_I _in the reduced Sub-Sample the significant comparisons were: 1 vs 3 (P = 0.019), 1 vs 4 (P = 0.001), 2 vs 4 (P = 0.016).

Table 1 reports the average anthropometric characteristic of the Full sample along with the mean values over the two samples (the Full Sample and the reduced Sub-Sample) of the four insulin sensitivity indices in the four BMI-identified classes. The ANOVA analysis among patient groups resulted significant for 1/HOMA-IR, HOMA2 and for K_xgI _both in the whole sample (P < 0.001 for the K_xgI_, P = 0.002 for the 1/HOMA-IR and P = 0.001 for the HOMA2) and in the reduced sub-sample (P < 0.001 for the K_xgI_, P = 0.005 for the 1/HOMA-IR and P = 0.001 for the HOMA2). S_I _was significantly different in the four groups only when the reduced sub-sample was considered (P = 0.006) and not significantly different among groups on the whole sample (P = 0.297). Figure 5 summarizes the comparison between the average values of S_I _and K_xgI _in the four BMI patient groups.

### Comparison with the EHC results

**Figure 6:**
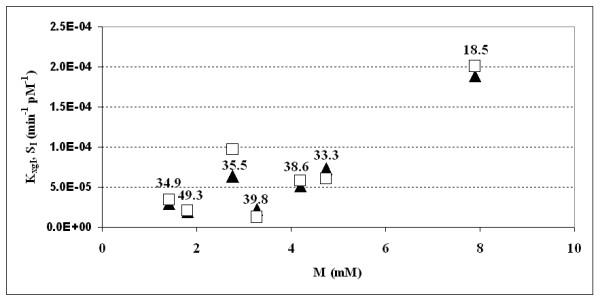
**Scatter plot of the two Insulin Sensitivity Indices (K_xgI _and S_I_) versus the M clamp-derived index of insulin sensitivity in seven subjects undergoing both IVGTT and Clamp**. Each couple of points has been labelled with the subject's BMI. Solid triangles = Single Delay Model K_xgI_, blank squares = Minimal Model S_I_.

Only 7 subjects were available in the present series, who also underwent an Euglycemic Hyperinsulinemic Clamp. On these, a further comparison was performed, given the widespread opinion that the EHC is the gold standard in the determination of insulin sensitivity. Figure 6 reports the values of the insulin sensitivity assessed with EHC (M index), along with the two insulin sensitivity indices, K_xgI _and S_I_: the two model-derived insulin sensitivity indices (K_xgI _and S_I _on the ordinate) are plotted against the clamp-derived insulin sensitivity M index (on the abscissa). It is to be noticed that these seven subjects happened to fall within the "good estimates" subgroup for the MM (S_I _CV < 52%). The points show a linear correlation between the two model-derived indices and the M. Given the small number of available subjects, the non parametric Spearman index was computed along with the parametric coefficient of correlation (Pearson's r). When the non parametric correlation is considered the P values are not significant, even if for the K_xgI _the P value is borderline (Spearman's rho = 0.75, P = 0.052 for the correlation K_xgI_-M; Spearman's rho = 0.571, P = 0.181 for S_I_-M); when the Pearson's r coefficient is computed both correlations result positive and significant (Pearson's r = 0.918, P = 0.004 for K_xgI _and Pearson's r = 0.832, P = 0.020 for S_I_). A thorough study is clearly necessary, involving a larger number of subjects.

### Relationship between the AIR and the K_xgI_

**Figure 7:**
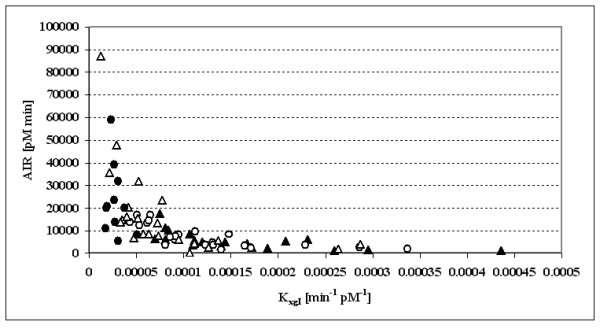
**Relationship between the SDM Insulin Sensitivity Index (K_xgI_) and the Acute Insulin Response (AIR = I_ΔG_/K_xgI_) in the 74 subjects**.

In order to evaluate the ability of the SDM to reproduce known physiologic relationships, the Acute Insulin Response (AIR) was computed [27,28] as the ratio of the difference of estimated initial condition and observed basal insulin (I_Δ _= I_0_-I_b_), over the first order insulin disappearance rate (AIR = I_Δ_/K_xi_). Figure 7 shows the scatter plot of available subjects over the SDM-K_xgI _and Acute Insulin Response plane. A one-way ANOVA test on AIR, with factor the BMI class, resulted significant (P = 0.001). The average values in the four classes were: 5666 ± 4053 for BMI ≥ 24, 7519 ± 5077 for 24 < BMI ≤ 30, 17069 ± 19690 for 30 < BMI ≤ 40 and 22956 ± 15606 for BMI > 40. The Disposition Index D_I _(computed as the product between AIR and K_xgI_) resulted instead not significantly different among the four BMI classes by one-way ANOVA (P = 0.718, average values: 0.69 ± 0.32 for BMI ≥ 24, 0.68 ± 0.25 for 24 < BMI ≤ 30, 0.76 ± 0.44 for 30 < BMI ≤ 40 and 0.61 ± 0.39 for BMI > 40).

A linear regression was also performed to evaluate whether the increase in AIR is linked to an increase in BMI: the beta coefficient was positive (β = 764) and significant (P < 0.001).

## Discussion

In the quest for simpler and more effective methods to evaluate the degree of sensitivity to insulin, the Intra-Venous Glucose Tolerance Test (IVGTT) has been proposed as an alternative to the established, but undoubtedly cumbersome, Euglycemic Hyperinsulinemic Clamp (EHC). The IVGTT-generated data, however, need to be interpreted by fitting onto them a suitable mathematical model: in the choice of the model to be applied, the possibility of reliably and precisely estimating an index of insulin sensitivity should be a major consideration, together with physiological plausibility, if the model is to be really useful to the diabetological community.

The aim of the present work is to evaluate a recently published model (the Single Delay Model, SDM) [13] for the glucose and insulin concentrations observed during a standard IVGTT, by applying it to a heterogeneous population composed of lean, overweight, obese and morbidly obese subjects. A further goal is to compare the SDM-derived insulin sensitivity index K_xgI _with the well known S_I _from the Minimal Model (MM).

The SDM, as presented in this work and as appeared in previous publications [13,15], was selected from a group of four two-compartment models, which differed according to the presence or absence of an insulin-independent glucose elimination rate term and according to the presence or absence of an explicit delay term for the action of insulin in stimulating tissue glucose uptake [13].

It is widely accepted that the observed effectiveness of insulin in producing appreciable decrease in glycemia lags behind the corresponding increase in insulinemia [28]. Explanations of this phenomenon may include the fact that interstitial insulin, rather than serum insulin, is responsible for glucose disappearance. The delay in the appearance of the insulin effect, besides being produced by the progressive (rather than instantaneous) lowering of glucose by tissues when stimulated by the hormone, may also depend on a specific delay of insulin action on those tissues. This delay in tissue insulin stimulation (which could stem from insulin distribution from plasma into interstitial space) can be mathematically represented by using either an unknown quantity (a further state variable representing an intermediate compartment, as in the MM, or even by a chain of similar added compartments) or by incorporating an explicit delay (discrete, distributed, etc.) in the action of serum insulin (which has to be transferred to the interstitium before exerting its effects). This last mathematical formulation allows the experimental assessment of whether such delay is indeed significantly different from zero, or whether it is relatively small and therefore practically negligible for data fitting. In fact, there is little doubt as to the fact that insulin needs to be transferred from plasma to, say, muscle cell surface, in order to produce its action. On the other hand, the actual time needed for this to happen may well be relatively small (if we think, for instance, that recirculation time is of the order of 1 minutes, including passage through both peripheral and lung capillaries, compared with the relatively long delay in lowering glycemia (which may be appreciated in tens of minutes). The comparison of models conducted in order to select the final form of the SDM showed that no explicit delay term was necessary for fitting available IVGTT data, which does not mean, as discussed before, that a delay does not exist.

The same can be said regarding the lack of a "glucose effectiveness term", i.e. of a first-order, insulin-independent tissue glucose uptake term. There appears in fact to be no normal physiological mechanism to support first-order glucose elimination from plasma: tissues in the body, except for brain, do not take up glucose irrespective of insulin; brain glucose consumption is relatively constant, and is subsumed, for the purposes of the present model, in the constant (zero-order) net hepatic glucose output term. A mass effect could indeed exist in the case when glycemias are above the renal threshold, where urinary glucose elimination, roughly proportional to above-threshold glycemias, is observed; and in the case when diffusion of glucose between compartments takes place. It must be emphasized that none of the subjects studied exhibited sustained, above-renal-threshold glycemias and that the rate of transfer attributable to plasma/interstitium equilibration (given again the observed circulation time of about two minutes) is much faster than what would be needed for insulin-independent tissue glucose uptake to contribute to the observed glycemia time course (with variations in the order of half-hours). A further substantial observation, against compartment equilibration playing a major role, is the estimated value of the volume of distribution for glucose, around 0.16 L/KgBW, comprising therefore interstitial water together with plasma volume. For all these reasons it would seem that no actual physiological mechanism would support the inclusion of an insulin-independent tissue glucose uptake term for the purpose of modeling the present series of subjects. It was in fact observed that, even if such a first-order mechanism were indeed present, its explicit representation did not prove necessary for the acceptable fitting of the present data series.

In future analyses it may however well be necessary to reintroduce insulin action delay or first order insulin-independent glucose uptake or both to explain observations under different conditions.

In the present series the two indices were compared also with the 1/HOMA-IR, the HOMA2 and (over a subsample) with the clamp-derived "M" index of insulin sensitivity.

The first result of the present assessment is that while in 50% of the subjects, the MM-derived S_I _is not significantly different from zero, and while several subjects exhibit questionably large or small S_I _values, the SDM-derived index of insulin sensitivity, K_xgI_, exhibits estimates with coefficient of variation less than 52% in every subject except one (whose estimated CV is in any case 69%) and with actual values covering a reasonable range (1.25 × 10^-5 ^to 4.36 × 10^-4^).

This result points to a marked degree of variability in the estimation of the parameters of the MM, compared with a very good numerical stability in the corresponding estimation of the SDM parameters. The instability of the S_I _index appears clearly also when considering correlation with the HOMA indices: it runs out when extreme S_I _values are considered, while it still persists between HOMA and K_xgI_. Reasons for this different behaviour have been discussed elsewhere [13], and can be summarised as a mathematical formulation more respectful of physiological understanding, of a smaller number of free parameters (the SDM is in fact more "minimal" than the MM because it fits both glycemias and insulinemias simultaneously using six free parameters instead of at least eight for the MM, having therefore a larger ratio of observations to estimable parameters), and in the avoidance of the statistically incorrect procedure of assuming interpolated noisy insulin concentrations as the true forcing function for glucose kinetics. Figures 2 and 3 show the performance of the two models in terms of their ability to describe the observed data. The apparent better fit of the Minimal Model is discussed at a great level of detail in [13]. Briefly, by using interpolated noisy observations as model input, the Minimal Model exploits the random variations of a single realization of the insulin kinetics to adapt coefficients in order to retrieve observed characteristics of the time course of glycemia. When fitting simultaneously glycemias and insulinemias, the Minimal Model (integrating Toffolo's [18] equation with the Bergman's original equations [11], see for example [14]) loses its ability to do so, fits more poorly than the SDM, and in fact loses the ability to reproduce the secondary insulin secretion phase 'hump'. Notice that in Figure 2 both insulin and glucose equations are fitted onto the data, while in figure 3 the insulin data are merely linearly interpolated. Finally, while the close adaptation to the data is certainly an important requisite of a good model, it is certainly not the primary consideration. If it were so, then polynomial or spline approximations would systematically outperform mechanistic models. The point is to find a simple mechanistic model, whose elements have a direct biological meaning, which closely fits available data, and the qualitative behaviour of whose solutions is compatible with physiology. For a critique to the Minimal Model from this point of view see [29].

There remains however the concern that, whatever the sophistication of the model, the well-known variability of insulin clearance makes it so that no insulin secretion analysis based on insulin levels alone can be expected to be fully accurate. It would be helpful to validate the results obtained for insulin secretion from the SDM against some gold standard indices of insulin secretion: indices based on C peptide measurement and the reconstruction of prehepatic insulin profiles could in fact be a possible candidate. However, not only the present data series, available to us, did not include C-peptide measurements for all 74 subjects, but the very deconvolution methods proposed so far in the literature to address this issue rely, themselves, on *ad-hoc *assumptions: one such being, e.g., the threshold based identification of the number of peaks from noisy C-peptide observed concentrations [30]. This problem deserves further study.

Even without considering the possible fitting of insulin observations to obtain information about the pancreatic response to circulating glucose, and limiting therefore the discussion to the estimation of insulin sensitivity by fitting glycemias, there are some problems in the standard approach. One is the phenomenon of the "zero-S_I_" [12], but even more important from a practical viewpoint is the large fraction of extreme estimates of the S_I _(18 out of 74, or 24.3% in the present series) and more generally of estimates of S_I _whose confidence interval contains zero, and to which therefore no meaningful estimate can be attributed (about 50% in the present series). Several recent publications [31-33] have addressed the improvement of estimation methods for the Minimal Model. The contention in the present work is that once the model itself is improved, then standard estimation methods are sufficient to obtain precise estimates. Furthermore, better estimation procedures, bayesian approaches, or population methods could be used for any model, for the SDM as well as for the Minimal Model.

The second result of the present work concerns the physiological correctness of the obtained estimates. While, in principle, estimates could be precise but biased, this in fact does not seem to be the case for the K_xgI _index as shown by the actual range of values, by the correlation with the 1/HOMA-IR and HOMA2 indices, by the correlation with the M index from the EHC, and by the very correlation with the S_I_, when questionable S_I _values are excluded. In fact, when excluding the 18 extreme S_I _values, the correlation S_I_-K_xgI _is very high and significant, and furthermore the Bland and Altman procedure shows the two measures to be equivalent.

While the S_I _suffers from the presence of questionable and extreme values, the K_xgI _correlates uniformly, and better than the S_I_, with the 1/HOMA-IR, HOMA2 and with the clamp-derived M-index over all available subjects. The limited size of the available sample of subjects who underwent both clamp and IVGTT does however represent a limitation of the present study, which should be addressed in the future by applying the SDM to other series of subjects simultaneously studied with both EHC and IVGTT.

The performance of the K_xgI _index has also been tested with regards to its ability of reproducing the well-known existing relationship between insulin resistance and body mass index. This is clearly visible in Table 1, where the considered population has been divided into four BMI subpopulations. Table 1 shows that increasing BMI is accompanied by a gradual decrease in insulin sensitivity, as estimated by either 1/HOMA-IR and HOMA2 or K_xgI _(in the full sample) or by S_I _(in the reduced sample only). The ANOVAs performed on the K_xgI _and on the 1/HOMA-IR and HOMA2 highlight significant differences of insulin sensitivity among the four classes. This result is obtained both in the full and in the reduced samples. For the S_I _the ANOVA resulted significant only when the reduced sub-sample is considered.

The lack of correlation of any insulin sensitivity index with the HOMA at extremes of insulin sensitivity may in fact reflect a limit of validity of HOMA in these ranges of insulin sensitivity values. Since the accuracy of HOMA mostly relies on the ability of fasting insulin to mirror insulin resistance, in the extreme insulin sensitivity ranges (high or low, e.g. athletes and T2DM subjects) the overall approximately hyperbolic relationship of HOMA and insulin sensitivity appears as a (respectively horizontal and vertical) asymptote, and correlation between insulin sensitivity and HOMA in both extreme ranges is lost. If this were the explanation of the lack of correlation of the HOMA with the S_I_, such lack should be apparent also between HOMA and K_xgI_, which is not the case, the values of correlation between HOMA and K_xgI _being essentially the same whether including or excluding the extreme ranges. The facts that this behaviour is the same both for the HOMA and for the newer and more accurate HOMA2, and that the large variability of SI index values would in any case produce lack of correlation by itself, lead us to hypothesize that the cause of the lack of correlation of the S_I _with the HOMA is essentially due to unreliable estimation of the S_I _itself.

The increase in AIR with increasing BMI is consistent with the current consensus. In non-diabetic subjects, fasting insulin secretion increases with BMI in an approximately linear fashion [34]. Similar results are obtained after an oral load of 75 g of glucose where total insulin output over the 2 h following ingestion increases in linear proportion with BMI [34]. It is also well known that there is a hyperbolic relationship between early insulin secretion, measured e.g. by the Acute Insulin Response (AIR) index, and insulin action, as expressed by an insulin sensitivity index, which, in the present case, is the model parameter K_xgI_.

This hyperbolic relationship of AIR with insulin sensitivity is well reproduced using the obtained SDM parameter estimates (the corresponding graph based on the full sample of S_I _estimates is not shown, given the extreme values which the S_I _index takes in some subjects). While not offering anything new from the physiological viewpoint, the confirmation of this relationship gives further support to the stability and meaningfulness not only of the insulin sensitivity index K_xgI_, but also of other SDM parameters, the AIR index being obtained in this case by the model-estimated I_ΔG_and K_xi_.

The observation that no significant relationship exists between the Disposition Index and BMI indicates that in the present series no progression of disease is apparent, in the sense that all subjects, whatever their body composition, seemed adequately compensated.

## Conclusions

The present model is obviously not supposed to describe all possible mechanisms intervening in the fate of secreted insulin and glucose uptake, but intends, in the present form, to relate peripheral serum insulin concentrations (an index of the actual insulin concentrations in interstitium, portal system, target tissues etc.) to observed glucose kinetics. Its purpose is exactly the same as that of the original Minimal Model, i.e. to provide the diabetologist with a simple mathematical way to interpret the IVGTT, and the contention made here is that the new model improves our ability to compute a robust, precise index of insulin sensitivity.

## Competing interests

The authors declare that they have no competing interests.

## Authors' contributions

SP: mathematical modelling and model computation, statistical analysis, drafting of the manuscript; ADG: mathematical modeling, drafting of the manuscript; GM: drafting of the manuscript; data provision; All authors read and approved the final manuscript.

## References

[B1] Groop LC Insulin resistance: the fundamental trigger of type 2 diabetes Diabetes Obes Metab 1999 1 S1 S7 10.1046/j.1463-1326.1999.0010s1001.x 11220283

[B2] Mahler R Adler M Clinical review 102: Type 2 diabetes mellitus: update on diagnosis, pathophysiology, and treatment J Clin Endocrinol Metab 1999 84 1165 1171 10.1210/jc.84.4.1165 10199747

[B3] Fontbonne A Eschwege E Insulin and cardiovascular disease: Paris prospective study Diabetes Care 1991 14 461 469 10.2337/diacare.14.6.461 1864219

[B4] Ferrannini E Buzzigoli G Bonadonna R Giorico M Oleggini M Graziadei L Insulin resistance in essential hypertension N Engl J Med 1987 317 350 357 3299096 10.1056/NEJM198708063170605

[B5] Després J Lamarche B Mauriège P Cantin B Dagenais G Moorjani S Hyperinsulinemia as an Independent Risk Factor for Ischemic Heart Disease N Engl J Med 1996 334 952 958 10.1056/NEJM199604113341504 8596596

[B6] Kincaid-Smith P Hypothesis: obesity and the insulin resistance syndrome play a major role in end-stage renal failure attributed to hypertension and labelled 'hypertensive nephrosclerosis' J Hypertens 2004 22 1051 1055 10.1097/00004872-200406000-00001 15167435

[B7] Farrell G Larter C Nonalcoholic fatty liver disease: from steatosis to cirrhosis Hepatology 2006 43 S99 S112 10.1002/hep.20973 16447287

[B8] Meigs G Epidemiology of the insulin resistance syndrome Curr Diab Rep 2003 3 73 79 10.1007/s11892-003-0057-2 12643149

[B9] Peter P Nuttall S Kendall M Insulin resistance--the new goal! J Clin Pharm Ther 2003 28 167 174 10.1046/j.1365-2710.2003.00482.x 12795774

[B10] Ferrannini E Natali A Bell P Cavallo Perin P Lalic N Mingrone G Insulin resistance and hypersecretion in obesity. European Group for the Study of Insulin Resistance (EGIR) J Clin Invest 1997 100 1166 1173 10.1172/JCI119628 9303923 PMC508292

[B11] Bergman RN Ider YZ Bowden CR Cobelli C Quantitative estimation of insulin sensitivity Am J Physiol 1979 236 E667 E677 443421 10.1152/ajpendo.1979.236.6.E667

[B12] Ni TC Ader M Bergman EN Reassessment of glucose effectiveness and insulin sensitivity from minimal model analysis: a theoretical evaluation of the single-compartment glucose distribution assumption Diabetes 1997 46 1813 1821 10.2337/diabetes.46.11.1813 9356031

[B13] Panunzi S Palumbo P De Gaetano A A discrete Single Delay Model for the Intra-Venous Glucose Tolerance Test Theoretical Biology and Medical Modelling 2007 4 10.1186/1742-4682-4-35 17850652 PMC2072949

[B14] Pacini G Bergman RN MINMOD: a computer program to calculate insulin sensitivity and pancreatic responsivity from the frequently sampled intravenous glucose tolerance test Comput Methods Programs Biomed 1986 23 113 122 10.1016/0169-2607(86)90106-9 3640682

[B15] Palumbo P Panunzi S De Gaetano A Qualitative behavior of a family of delay-differential models of the glucose-insulin system Discrete and Continuous Dynamical Systems - Series B 2007 7 399 424

[B16] Defronzo RA Tobin JD Andres R Glucose clamp technique: a method for quantifying insulin secretion and resistance Am J Physiol 1979 237 E214 E223 382871 10.1152/ajpendo.1979.237.3.E214

[B17] Davidian M Giltinan DM Nonlinear Models for Repeated Measurement Data 1995

[B18] Toffolo G Bergman RN Finegood DT Bowden CR Cobelli C Quantitative estimation of beta cell sensitivity to glucose in the intact organism: a minimal model of insulin kinetics in the dog Diabetes 1980 29 979 990 10.2337/diabetes.29.12.979 7002673

[B19] Press WH Flannery BP Teukolsky SA Vetterling WT Numerical recipes in C. The art of scientific computing 1994 2 Cambridge: Cambridge University Press

[B20] Malita F Karelis A St-Pierre D Garrel D Bastard J Tardif A Surrogate indexes vs. euglycaemic-hyperinsulinemic clamp as an indicator of insulin resistance and cardiovascular risk factors in overweight and obese postmenopausal women Diabetes & Metabolism 2006 32 251 255 10.1016/s1262-3636(07)70276-8 16799402

[B21] Ciampelli M Leoni F Cucinelli F Mancuso S Panunzi S De Gaetano A Assessment of insulin sensitivity from measurements in the fasting state and during an oral glucose tolerance test in polycystic ovary syndrome and menopausal patients J Clin Endocrinol Metab 2005 90 1398 1406 10.1210/jc.2004-0410 15598698

[B22] Matthews DR Hosker JP Rudenski AS Naylor BA Treacher DF Turner RC Homeostasis model assessment: insulin resistance and beta-cell function from fasting plasma glucose and insulin concentrations in man Diabetologia 1985 28 412 419 10.1007/BF00280883 3899825

[B23] Emoto M Nishizawa Y Maekawa K Hiura Y Kanda H Kawagishi T Homeostasis model assessment as a clinical index of insulin resistance in type 2 diabetic patients treated with sulfonylureas Diabetes Care 1999 22 818 822 10.2337/diacare.22.5.818 10332688

[B24] Bonora E Targher G Alberiche M Bonadonna R Saggiani F Zenere MB Homeostasis model assessment closely mirrors the glucose clamp technique in the assessment of insulin sensitivity: studies in subjects with various degrees of glucose tolerance and insulin sensitivity Diabetes Care 2000 23 57 63 10.2337/diacare.23.1.57 10857969

[B25] Yokoyama H Emoto M Fujiwara S Motoyama K Morioka T Komatsu M Quantitative Insulin Sensitivity Check Index and the Reciprocal Index of Homeostasis Model Assessment in Normal Range Weight and Moderately Obese Type 2 Diabetic Patients Diabetes Care 2003 26 2426 2432 10.2337/diacare.26.8.2426 12882874

[B26] HOMA Calculator The Oxford Centre for Diabetes Endocrinology & Metabolism Diabetes Trial Unit 2009

[B27] Kahn SE Prigeon RL McCulloch DK Boyco EJ Bergman RN Schwartz MW Quantification of the relationship between insulin sensitivity and b-cell function in human subjects Diabetes 1993 42 1663 1672 10.2337/diabetes.42.11.1663 8405710

[B28] Bergman RN Lilly lecture 1989. Toward physiological understanding of glucose tolerance. Minimal-model approach Diabetes 1989 38 1512 1527 10.2337/diabetes.38.12.1512 2684710

[B29] De Gaetano A Arino O Mathematical modelling of the intravenous glucose tolerance test J Math Biol 2000 40 136 168 10.1007/s002850050007 10743599

[B30] Van Cauter E Estimating false-positive and false-negative errors in analyses of hormonal pulsatility American Journal of Physiology-Endocrinology and Metabolism 1988 254 E786 E794 10.1152/ajpendo.1988.254.6.E786 3377077

[B31] Cobelli C Caumo A Omenetto M Minimal model SG overestimation and SI underestimation: improved accuracy by a Bayesian two-compartment model Am J Physiol Endocrinol Metab 1999 277 481 488 10.1152/ajpendo.1999.277.3.E481 10484360

[B32] Pillonetto G Caumo A Sparacino G Cobelli C A new dynamic index of insulin sensitivity Ieee Transactions on Biomedical Engineering 2006 53 369 379 10.1109/TBME.2005.869654 16532763

[B33] Pillonetto G Sparacino G Cobelli C Numerical non-identifiability regions of the minimal model of glucose kinetics: superiority of Bayesian estimation Math Biosci 2003 184 53 67 10.1016/S0025-5564(03)00044-0 12788233

[B34] Ferrannini E Camastra S Gastaldelli A Sironi A Natali A Muscelli E Beta-Cell Function in Obesity. Effects of Weight Loss Diabetes 2004 53 S26 S33 10.2337/diabetes.53.suppl_3.S26 15561918

